# Personalized Rehabilitation Recognition for Ubiquitous Healthcare Measurements

**DOI:** 10.3390/s19071679

**Published:** 2019-04-08

**Authors:** Yao-Chiang Kan, Yu-Chieh Kuo, Hsueh-Chun Lin

**Affiliations:** 1 Department of Electrical Engineering, Yuan Ze University, Chung-Li, Taoyuan City 32003, Taiwan; yckan@saturn.yzu.edu.tw; 2 Department and Institute of Health Service Administrations, China Medical University, Taichung 40402, Taiwan; fish840319@gmail.com

**Keywords:** adaptive neuro-fuzzy inference system, physiotherapy exercise, rehabilitation recognition, sensor-enabled wristband, ubiquitous healthcare measurement

## Abstract

The physical therapeutic application needs personalized rehabilitation recognition (PRR) for ubiquitous healthcare measurements (UHMs). This study employed the adaptive neuro-fuzzy inference system (ANFIS) to generate a PRR model for a self-development system of UHM. The subjects wore a sensor-enabled wristband during physiotherapy exercises to measure the scheduled motions of their limbs. In the model, the sampling data collected from the scheduled motions are labeled by an arbitrary number within a defined range. The sample datasets are referred as the design of an initial fuzzy inference system (FIS) with data preprocessing, feature visualizing, fuzzification, and fuzzy logic rules. The ANFIS then processes data training to adjust the FIS for optimization. The trained FIS then can infer the motion labels via defuzzification to recognize the features in the test data. The average recognition rate was higher than 90% for the testing motions if the subject followed the sampling schedule. With model implementation, the middle section of motion datasets in each second is recommended for recognition in the UHM system which also includes a mobile App to retrieve the personalized FIS in order to trace the exercise. This approach contributes a PRR model with trackable diagrams for the physicians to explore the rehabilitation motions in details.

## 1. Introduction

Rehabilitation is an important scope of physiotherapy for patients healing from severe injuries such as paralysis, hemiplegia, handicaps, surgery, etc. Physical therapists design specific exercises to extend the range of motion (ROM) of joints to moderately improve the flexibility of limbs [1,2]. A rehabilitation exercise usually assembles diverse joint kinematics, including flexion, extension, abduction, adduction, pronation, supination, rotation, and deviation regarding the disabled part [3,4,5]. Many studies and clinical prescriptions have thus suggested routine exercises for rehabilitation in healthcare services [6,7]. Modern hospitals offer the necessary functional exercise therapy facilities to serve the patients with scheduled programs [8,9]. In addition, physiatrists will have concerns about the performance of exercises and relevant joint motions, which need to be practiced daily practice at home to manage a health promotion program [10,11].

Present healthcare services promote ubiquitous healthcare measurements (UHMs) for the self-management of those patients who need regular rehabilitation at home. Non-imaging detection is an important ethical issue regarding monitoring handicapped patients in rehabilitation healthcare [12,13]. Physiatrists can assign a program of specific motions for those patients needing routine exercises during their recovery period; then, they are able to track the daily records of patients via the UHM system for the purpose of prescribing advanced therapies [14]. Rehabilitation UHMs are obtained via body sensor networks (BSNs), which provide a wireless sensor network (WSN) of wearable computing devices within a certain area, to continuously log in the motion data of patients [15,16,17]. With the BSN, the UHM requires data quality, management interface, sensor validation, cost, data consistency, constrained devices, interoperability, etc. [18] Thus, wristbands embedded with an accelerometer and gyroscope have been widely used to detect the body movements of people while sleeping, falling, walking, exercising, cleaning, etc. [19]. In recent years, these sensor-embedded wearable devices have become popular among people during their daily activities [20,21]. A sensor-enabled wristband and a smart phone can be implemented in the BSN to transmit the measurement data for UHM requirements through the Bluetooth low energy (BLE) protocol, which provides reduced-power consumption and cost within a similar communication range as classic Bluetooth [22]. Thus, the rehabilitation exercise data can be measured by a wristband embedded with a BLE-based sensor for UHM [23,24]. With the proper algorithms, a personalized rehabilitation recognition (PRR) pattern of a patient is trackable for management [25].

Many computing algorithms have been employed in the study of activity recognition. In general, the artificial neural network (ANN), backward propagation neural network (BPNN), fuzzy logic theory, etc. are well-known algorithms for classifying and recognizing motion features [26]. For instance, the ANN retains training ability to learn complicated movements [27]; the BPNN holds three layers in machine learning to categorize activities [28]; and the rule-based fuzzy inference system (FIS) supports stationary patterns for measuring a regular motion tendency [29,30,31]. In FIS, a fuzzy set of possible features is assembled by the membership function (MF) for fuzzification from input to output, and then a list of if-then rules is utilized for controlling the defuzzification process [32]. In terms of defuzzification, the Mamdani and Sugeno models are two major types that present the MFs and linear-expression crisps (or constant), respectively, for the output features in the FIS [33,34]. For inferring the output levels, the Mamdani model computes the centroid of the union area of the MFs, whereas the Sugeno model entails computing the weight average of the crisps [35]. In addition, the adaptive neuro-fuzzy inference system (ANFIS) that repetitively tunes the FIS in a training-based algorithm has been suggested to optimize the inference ability of the adopted features [36,37]. Therefore, the FIS can be one of the appropriate methods to recognize the human activity for healthcare measurement since the activity subject performs the characteristics of behavior with the inferable features [38,39].

This study proposes a PRR model based upon upper-limb kinematics by integrating the ANFIS with rule-based fuzzy logic and a data training process to meet UHM requirements. The model extends our previous work on WSN measurement of general human activities (e.g., sitting, standing, lying, walking, running etc.) [39] to recognize motions of rehabilitation exercises using a BLE-compliant wearable sensor “MetaWearC” by MbientLab, Inc. [40]. The commercial sensor including accelerometer and gyroscope chips was installed in an assembled wristband for convenient data collection, whereas the released developer kits were employed to create adaptable mobile Apps in a smart phone. The ANFIS toolboxes by MATLAB^TM^ were used in modeling a FIS engine, and then a self-developed FIS App was compiled using the open-source FuzzyLite library [41] to drive the engine in a UHM system for calibrating the PRR datasets. In this paper, we have organized the sections as follows: the Methods and Modeling section describes the design of the PRR exercise, introduces the ANFIS algorithm, and constructs the UHM framework. The Results and Evaluation section reveals the outcome and evaluates the recognition accuracy. Consequently, the Implementation and Discussion section proves the adaptability of the model and discusses its feasibility. Finally, the Conclusions section summarizes the findings.

## 2. Methods and Modeling

The proposed PRR model applies the ANFIS based on fuzzy inference theory for machine learning. The scheduled exercises were designed for the wearable sensor to achieve the ubiquitous healthcare measurement by recognition computing; in which, the chip of the BLE-compliant BMI160 with a 6-axis accelerometer and gyroscope [42] is embedded in the sensor to detect accelerations and angular velocities of motions about three axes. Thus, the components of tilt angle vector can be calculated by the acceleration vector. The sensor can send signals at frequencies from 20 to 100 Hz; i.e., the sensing frequency is adjustable to collect 20~100 data per second. For instance, if the exercise is scheduled by 1 min and the frequency is controlled at 100 Hz, then 6000 raw datapoints can be collected. Each raw datapoint will contain six components of the angular velocity and acceleration in the x, y, and z axes, which can be further derived into the datasets of candidate features such as relative angles, angular velocities, accelerations with respect to the origin position of the sensor, etc.

### 2.1. Rehabilitation Motion Design

**Figure 1:**
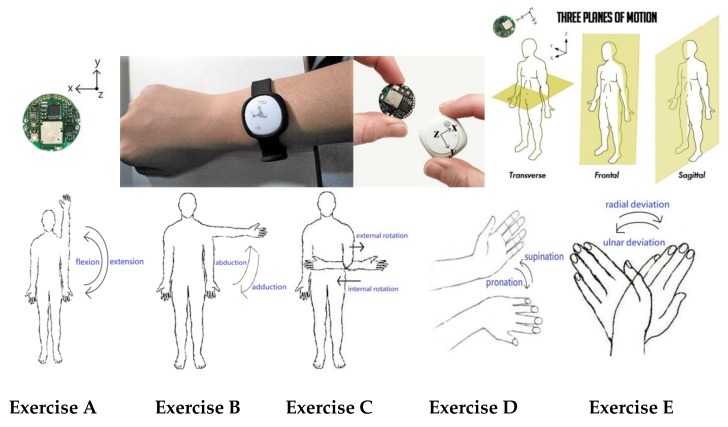
Wristband with BLE-compliant sensor corresponding to local and global coordinates, and the designed simple exercises: (**A**) flexion-extension, (**B**) abduction-adduction, (**C**) external-internal rotation, (**D**) pronation-supination, (**E**) ulnar-radial deviation.

The subject wears a sensor-embedded wristband on their wrist for the measurements. We define the neutral position of the human-body as the global coordinate (i.e., X, Y, Z axes orthogonal to the frontal, sagittal, and transverse planes of the human body, respectively), whereas the center of the sensor presents local coordinates (i.e., x, y, and z axes toward to the side, band, and top of the wristband) as shown in Figure 1. For measuring the exercise, the motion is designed in the global coordinates, but the sensor returns signals in local coordinates. Thus, we can calibrate the measured data with respect to the origin, which is the initial position of the sensor, upon starting the exercise.

In the essential rehabilitation of the upper limbs, the exercises usually include motions of extension, flexion, abduction, adduction, rotation, and deviation to improve the ROM of the joints at the shoulder, elbow, and wrist. In this study, we took some typical ROM exercises using the guidelines below as examples to design the sampling schedule of motions for the proposed model:

*Exercise A*. Flexion and extension (flex-ext) of shoulder: wear the wristband on the wrist, straighten the arm downward and place the palm facing backward for the initial state; (1) keep the initial state for 2 s, (2) slowly raise the arm up (flexion) to the head in 4 s, (3) hold the limb above for 2 s, (4) slowly drop the arm down (extension) to the initial state in 4 s, then a cycle has been completed; repeat this cycle five times.

*Exercise B*. Horizontal abduction and adduction (abd-add) of shoulder: wear the wristband on the wrist, straighten the arm downward and place the palm facing inward for the initial state; (1) keep the initial state for 2 s, (2) laterally raise the arm up to shoulder level (abduction) in 4 s, (3) hold the limb at shoulder level for 2 s, (4) slowly drop the arm down (adduction) to the initial state in 4 s, then a cycle has been completed; repeat this cycle five times.

*Exercise C*. External and internal rotation (ext-int rot) of elbow: wear the wristband on the wrist, bend the elbow 90° with upper arm always close to the body, and place the palm inward facing the abdomen for the initial state; (1) keep the initial state for 2 s, (2) slowly rotate the arm away from facing inward toward the abdomen to facing outward in 4 s (external rotation), (3) hold the limb there for 2 s, (4) slowly rotate the arm toward the abdomen in 4 s (internal rotation), then a cycle has been completed; repeat this cycle five times.

*Exercise D*. Pronation and supination (pron-supin) of elbow and wrist: wear the wristband on three fingers, bend the elbow 90° with the upper arm always close to the body, and with the palm facing upward for initial state; (1) keep the initial state for 2 s, (2) slowly rotate the forearm into a palm-downward position in 4 s (pronation), (3) hold the limb there for 2 s, (4) slowly rotate the forearm into a palm upward position (supination) in 4 s, then a cycle has been completed; repeat this cycle five times.

*Exercise E*. Ulnar and radial and deviation (ulnar-rad dev) of wrist: wear the wristband on three fingers and put the palm on a desk or table for the initial state; (1) keep the initial state for 2 s, (2) slowly bending the wrist to the little finger side (ulnar deviation) in 4 s, (3) hold the limb there for 2 s, (4) slowly bend the wrist to the thumb side (radial deviation) in 4 s, then a cycle has been completed; repeat this cycle five times.

In this study, we took *Exercise A* as an example to describe the proposed recognition process. The five simple exercises with joint motions mentioned above were combined in the union and complex exercises for practice, and this is further discussed in the Implementation section. The motions are labeled by numbers and their definitions are shown in Table 1, which exhibits the abbreviation of each motion and the numerical range of the motion label.

### 2.2. Modeling

**Figure 2:**
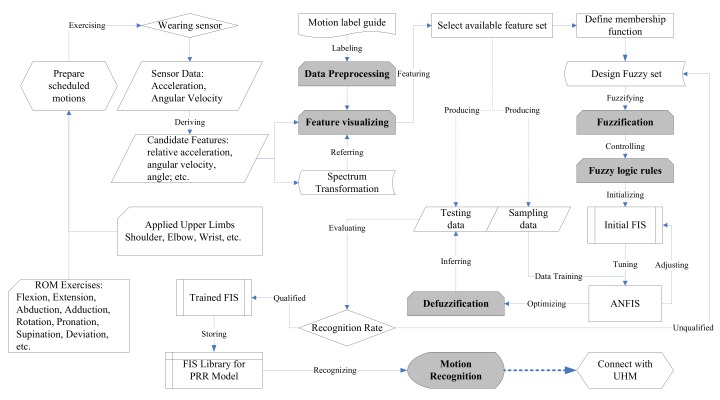
ANFIS computing flowchart for the PRR model.

The modeling process follows five major steps: (1) data preprocessing and sampling, (2) feature visualizing, (3) fuzzification, (4) fuzzy logic rule and data training, and (5) defuzzification, prior to generate a proper FIS for personalized rehabilitation recognition. Figure 2 illustrates the computing flowchart of the modeling process above.

#### 2.2.1. Data Preprocessing and Sampling

According to the designed exercise, the subject wore the wristband and repeated the scheduled motions to produce a sample dataset. Using the example of *Exercise A*, the motions were labeled by the arbitrary numbers ranging in [0, 1), [1, 2), [2, 3), and [3, 4), respectively, for the steps (1), (2), (3), and (4) of the flexion-extension exercise as shown in Table 1. In the physiotherapy, the subject should finish a therapeutic exercise with the correct motions based on the schedule. The unsupervised data of the personalized motions were labeled by a standard schedule as the sample dataset for supervised machine learning. Additionally, the subject followed the same schedule in order to produce a test dataset for evaluation.

We can preprocess the measured data using a fuzzy algorithm from our previous study, which suggested the transformation process to select the possible features [39]. The features include the relative acceleration, angular velocity, and angle of the motion with respect to the original position of the wristband. Table 2 shows the candidate features and their abbreviations in computing. With the exercise schedule, the labeled sample data are available for featuring in the initial FIS and supervised machine learning in the ANFIS procedure.

#### 2.2.2. Feature Visualizing

**Figure 3:**
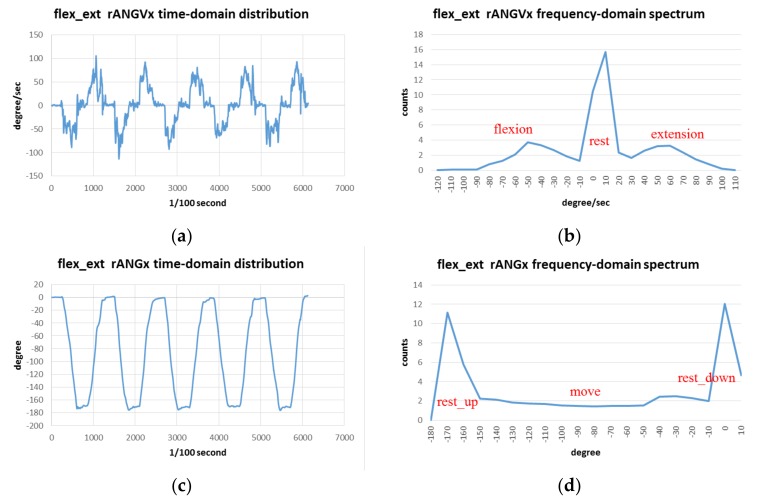
Measured sampling data transform between time-domain distribution and frequency-domain spectrum, as exemplified by the input features of the flex-ext exercise: (**a**) Time-domain distribution and (**b**) Frequency-domain spectrum of sampling relative angular velocities about x axis; (**c**) Time-domain distribution and (**d**) Frequency-domain spectrum of sampling relative angles about x axis.

The available features can be adopted for fuzzy computing through the visualized diagrams. The data distribution, which was scheduled in time domain, can be transformed to frequency domain (or a spectrum) as shown in Figure 3. With visualization, the spectra are in relation to the available features that can help with computing the corresponding motions. For an example of the relative angle on the x axis (rANGx), the amount of moving angles measured in the flex-ext exercise is plotted as a spectrum. The angles for holding the arm on the top and bottom positions are around 0° and −180°, respectively; while the angles of the raising-up and putting-down motions in this range vary with the relative angular velocities about the x axis (rANGVx). We can observe these spectra and refer them to create the membership function (MF) of the fuzzy set in the model.

**Figure A1:**
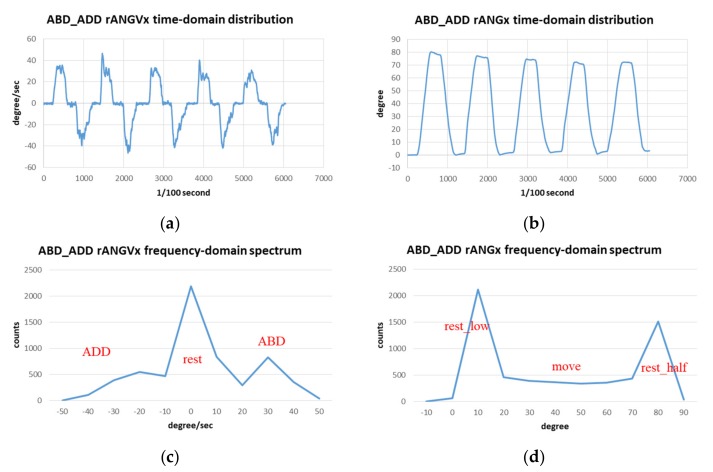
Input feature data transform for the exercise of horizontal abduction-adduction (abd-add): (**a**) Time-domain distribution and (**b**) Frequency-domain spectrum of relative angular velocities about x axis (rANGVx); (**c**) Time-domain distribution and (**d**) Frequency-domain spectrum of relative angles about x axis (rANGx).

**Figure A2:**
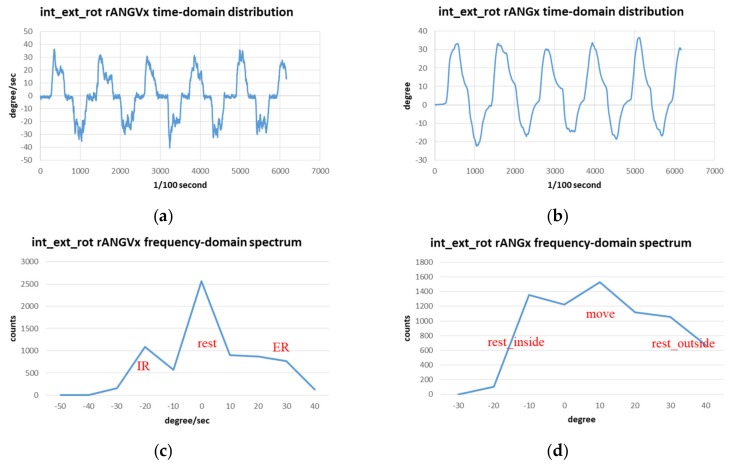
Input feature data transform for the exercise of internal-external rotation (int-ext rot): (**a**) Time-domain distribution and (**b**) Frequency-domain spectrum of relative angular velocities about x axis (rANGVx); (**c**) Time-domain distribution and (**d**) Frequency-domain spectrum of relative angles about x axis (rANGx).

**Figure A3:**
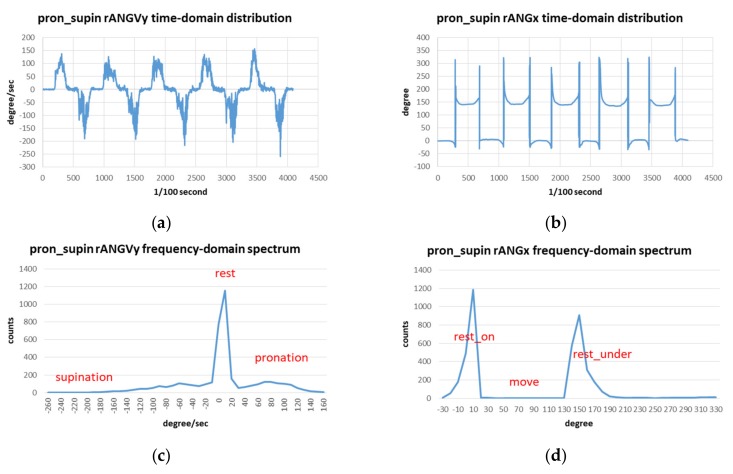
Input feature data transform for the exercise of pronation-supination (pron-supin): (**a**) Time-domain distribution and (**b**) Frequency-domain spectrum of relative angular velocities about y axis (rANGVy); (**c**) Time-domain distribution and (**d**) Frequency-domain spectrum of relative angles about x axis (rANGx).

**Figure A4:**
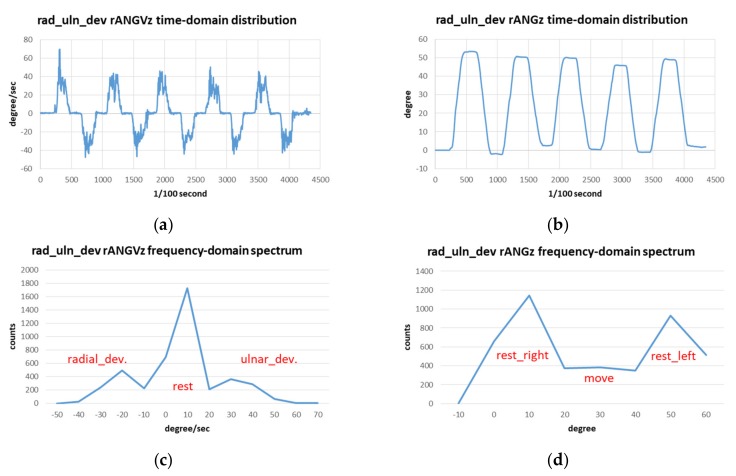
Input feature data transform for the exercise of radial-ulnar deviation (rad-uln dev): (**a**) Time-domain distribution and (**b**) Frequency-domain spectrum of sampling relative angular velocities about z axis (rANGVz); (**c**) Time-domain distribution and (**d**) Frequency-domain spectrum of relative angles about z axis (rANGz).

For this example, we selected rANGVx and rANGx as the features. Their diagrams as shown in Figure 3 perform the still and moving motions for the flex-ext exercise. In addition, other variables can also become the feature set depending on the characteristics of personal behaviors. Table 2 presents the chosen features for the exercises modeled in this study; while the transforms regarding *Exercises B, C*, *D*, and *E* are presented in Appendix A (Figure A1, Figure A2, Figure A3 and Figure A4, respectively).

#### 2.2.3. Fuzzification and Featuring

**Figure 4:**
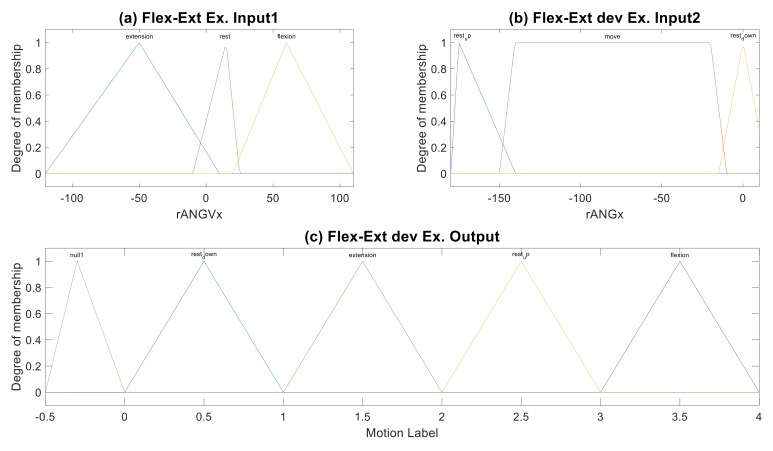
Fuzzy set example for the flex-ext exercise of shoulder: (**a**) Input1—membership functions of input feature “rANGVx”, (**b**) Input2—membership functions of input feature “rANGx”, (**c**) Output—Membership functions of output feature “Motion Label”.

Several types of the MF can be chosen to create the fuzzy set for a FIS, which is a process required prior to data training. We explored the typical MFs including the triangle, trapezoid, Gaussian, sigmoid, bell-shape, s-shape, z-shape, -shape functions, etc. to generate an initial FIS model for training the ANFIS. With reference to the spectra of rANGVx and rANGx variables in the example, we adopted the triangle and trapezoid MFs for the input features, and the triangle MFs for the output features as shown in Figure 4. In this case, the flex-ext exercise consists of four-step motions in a cycle and each motion is labeled by a random value in the range of [0, 1), [1, 2), [2, 3), and [3, 4) corresponding to the MFs of the output feature. The neighbor MFs of the output feature are capable of a state of motion changing; e.g., the motion labelled by 0.9 or 1.1 can be inferred to either step (2) “raising arm up” or (3) “holding on top”. Additionally, combination of the various input features with the proper MFs can yield a different fuzzy set for the same output motions.

The function-based MFs of output feature (i.e., Mamdani type) must be converted to the crisp set (i.e., Sugeno type) before the training process in the ANFIS. The module “mam2sug” in the fuzzy logic toolbox of MATLAB^TM^ is employed to adapt the functional MFs to the crisp value. As shown in Table 3, the MFs of input and output features for the flex-ext exercise include coefficients of the Mamdani-type functions and Sugeno-type equations. The triangle MF can be illustrated by a set of vertex coefficients, e.g., [–120,–50,10]; while the linear MF can be formulated by a set of equation coefficients, e.g., [0.0077, 0.1022, 0.913].

**Figure B1:**
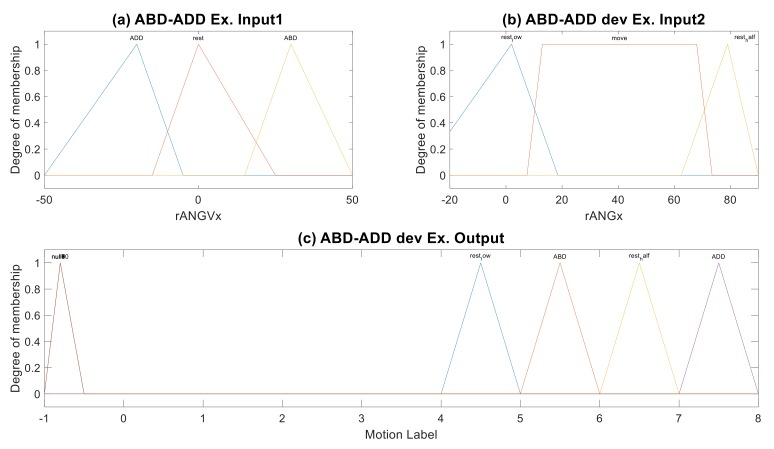
Fuzzy set for the abd-add exercise of shoulder: (**a**) Input1—membership functions of input feature “rANGVx”; (**b**) Input2—membership functions of input feature “rANGx”; (**c**) Output—membership functions of output feature “Motion Label”.

**Figure B2:**
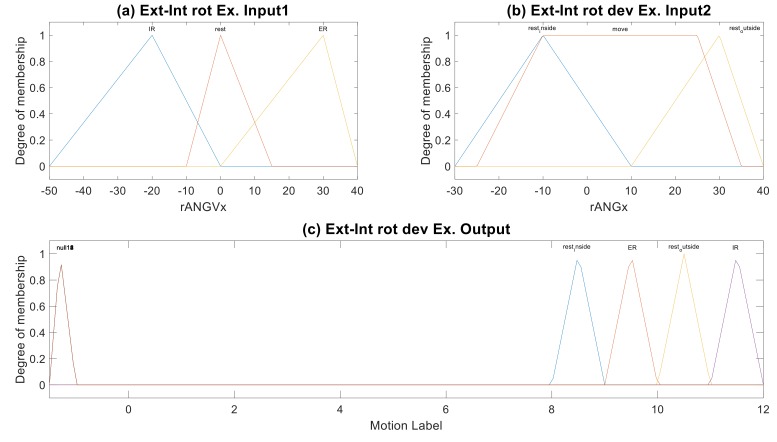
Fuzzy set for the ext-int rot exercise of elbow: (**a**) Input1—membership functions of input feature “rANGVx”; (**b**) Input2—membership functions of input feature “rANGx”; (**c**) Output—membership functions of output feature “Motion Label”.

**Figure B3:**
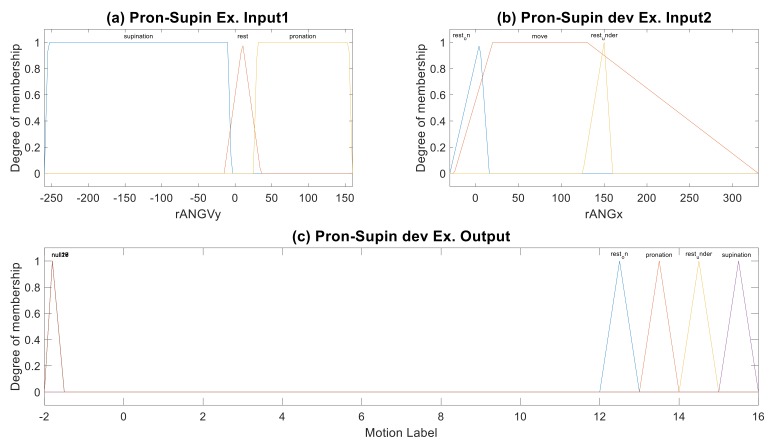
Fuzzy set for the pron-supin exercise of wrist: (**a**) Input1—membership functions of input feature “rANGVy”; (**b**) Input2—membership functions of input feature “rANGx”; (**c**) Output—membership functions of output feature “Motion Label”.

**Figure B4:**
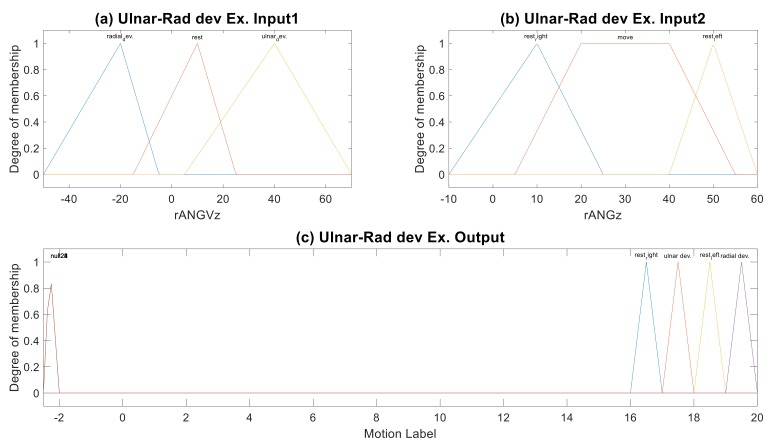
Fuzzy set for the ulnar-rad exercise of wrist: (**a**) Input1—membership functions of input feature “rANGVz”; (**b**) Input2—membership functions of input feature “rANGz”; (**c**) Output—membership functions of output feature “Motion Label”.

In addition, the amount of MFs in the output feature must be a multiplication of those in the input features for training in the ANFIS. The Sugeno-type fuzzy set in this example needs nine MFs (3 × 3 = 9) of the output feature in relation to the input features. We thus can create five more triangle MFs by a virtual label (e.g., [−0.5, 0) as shown in Figure 4c) for virtual motions in addition to four real motions. The fuzzy sets regarding *Exercises B, C, D* and *E* are provided in Appendix B (Figure B1, Figure B2, Figure B3 and Figure B4, respectively)

#### 2.2.4. Fuzzy Logic Rule and Data Training

In this cyclic step, a fuzzy logic rule is defined to control the relationship between the input and output features. The MFs of input features are assigned to the corresponding MFs of output based upon the rules. For instance, if the feature “rANGx” is around −180 degrees and “rANGVx” is around zero, then the motion label should be in [2, 3) (i.e., motion (3) “hold the arm on top”) due to Rule 3 as shown in Table 4. Thus, the fuzzy logic rule can be formatted as [if rANGVx is i_rest and rANGx is i_rest_up, then motion is o_rest_up]. For converting this type of Mamdani to Sugeno in this case, we created nine rules including five dummy rules corresponding to the virtual motions, which are never matched but required for the FIS structure.

In this cyclic step, an initial FIS is tuned by the ANFIS via a data training process, which adjusts the parameters of MFs by minimizing the root mean square errors (RMSEs) of the FIS repetitively for each epoch till an optimal status is reached (i.e., the error variation approaches stability). The output FIS after data training can be evaluated by the test datasets which are acquired from the subject following the same schedule. Table 3 shows the trained FIS of the Sugeno model, which optimizes the output level by a linear equation for recognition in the next step.

#### 2.2.5. Defuzzification and Recognition

**Figure 5:**
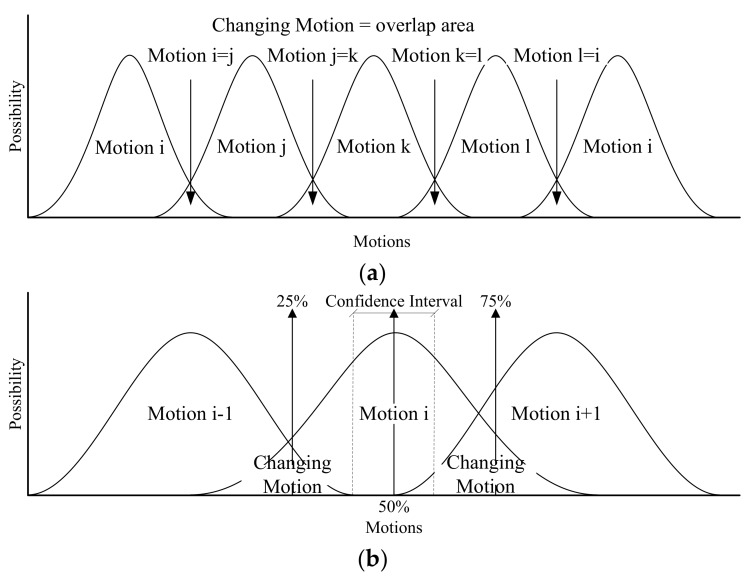
Recognition schemes: (**a**) the adaptable motion-label scheme allows the identical labels in changing motion, (**b**) the quartile motion-label scheme computes the confidence interval in a motion schedule.

The trained FIS includes the capable MFs and fuzzy rules to drive the defuzzification process and infer the motion labels in the test data. The process computes the union area of the MF curves due to their degrees of participation in the logic of the rules. The toolbox uses the centroid and weighted-average methods for the Mamdani- and Sugeno-type FIS, respectively. The inferred labels can be compared with the motion schedule for evaluation; i.e., they should be in the same label range if the sampling schedule is followed. We proposed the adaptable scheme and quartile schemes as shown in Figure 5, which analyze the adaptable and quartile motion labels for data extraction in practical measurement, respectively, to evaluate the recognition rate of the FIS.

The adaptable motion-label scheme recognizes an exercise excluding motion changes. For instance, the motion is probably at the state of “pause at the bottom” or “raise-up” when the subject begins to raise the arm. At that moment, the motion label defuzzified in either [3, 4) or [0, 1) is acceptable—i.e., the motion is correct if inferred as “pause” or “raise”. We adapted 10% of estimated dataset for the acceptable range as changing the motion.

The quartile motion-label scheme splits the data distribution of a motion into four sets (i.e., cut at 25%, 50%, 75%), and it evaluates the rate for each quartile set. The sets of 25% and 75% are usually counted as changing the motion, thus the middle portion (i.e., the data set around 50%) would be the confidence interval of the reliable motion labels. We can adopt the inferred motion labels within the reliable set to evaluate the recognition rate. The validated FIS can be further implemented in the UHM system to track the assigned physiotherapy exercises.

### 2.3. Ubiquitous Healthcare Measurement System

**Figure 6:**
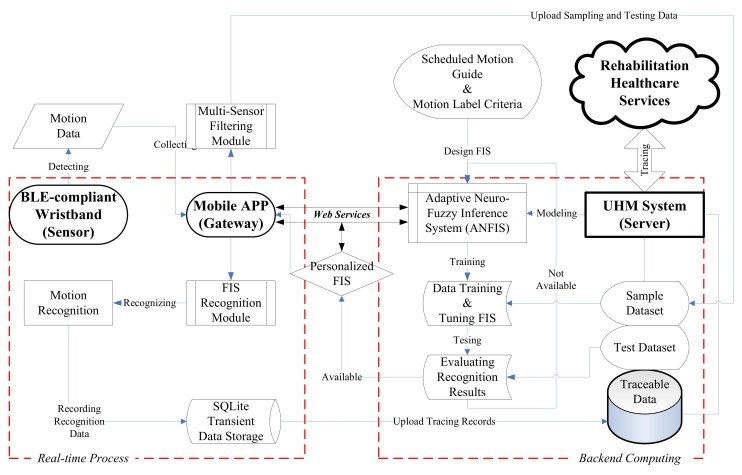
Architecture of the three-layer UHM system based upon the Internet of Things.

We established a prototype of the UHM system to implement the proposed PRR model. The system architecture with its data flowchart is shown in Figure 6, which according to the Internet of Things (IoT) involves three layers: the sensor, the gateway, and the server. In measurement, the smart phone can be the gateway to receive and transmit signals of the BLE sensor. The measured data in the gateway are transformed into the possible features such as relative angles, accelerations, angular velocities, etc., by mobile Apps; then, they are sent to the backend server via the Internet for data training in the server [43]. With the ANFIS, the UHM system serves the tuned FIS parameters for the self-developed App to recognize the physiotherapy exercises. The major components of the system architecture are addressed below.

We designed two multi-sensor filtering (MSF) and FIS recognition (FISR) modules in the mobile App with an user-friendly interface. The MSF module enhances the Android application program interface (API) of MbientLab for the MetaWearC sensor to acquire the signals from multiple sensors and filter out the relevant features. The FISR module involves the open-source API of FuzzyLite to drive the trained FIS for recognizing motions.

(A) *MSF module*. The MSF provides the objects of “SensorConnect,” “MotionAnalysis,” and “DataTransfer”. The “SensorConnect” implements the MbientLab APIs to connect with the sensors and retrieve the detected data. The “MotionAnalysis” processes the functions of featuring and transferring data to derive the candidate features due to the raw data of acceleration and angular velocity. The “DataTransfer” accesses the data between mobile and server databases as well as labels the sample data according to the motion guide and exercise schedule.

(B) *FISR module*. The FISR includes the objects of the “FISEngine” and “FISMotion” for Fuzzy computing. The “FISEngine” utilizes the FuzzyLite APIs to parse the FIS model and select the proper features to defuzzify the motion data. The “FISMotion” drives the “FISEngine” to compute the motion labels for recognition. In addition, the FISR module can also evaluate the accuracy of the test dataset in the server layer prior to feedback of the trained FIS.

In the training phase, the MSF module can receive the sample data (e.g., 100 data per second) from the wristband, extract the necessary features, and then upload to a measurement database in the UHM server for data training. Once the training procedure is completed, the trained FIS files are stored in a library of personalized criteria for recognition. In the recognition phase, the FISR module can download the FIS file via Web services to recognize the motions for tracking the exercise. The tracked data (e.g., one data per second) will be saved in SQLite storage (e.g., a memory card) and uploaded to health-promotion database for remote tracking. In practice, the subject can get an identical number of the personal exercise for the training step, and the App can offer an associated FIS for selection before recognition. With ANFIS computing, the PRR model can be practiced in a self-developed UHM system to manage the rehabilitation program.

## 3. Results and Evaluation

The test datasets of five ROM exercises were applied to evaluate the trained FIS. One hundred datasets were received in a second (i.e., the sensor frequency is 100 Hz), and five-cycles of an exercise was scheduled to produce thousands of sample datasets for training. Then, the subject followed the exercise schedule to the best of their ability to provide the test data, in which at least one cycle of motions satisfying the sampling procedure can be chosen for evaluation. Both schemes of the adaptable and quartile motion labels are employed to calculate the recognition rate.

### 3.1. Inference Result

**Figure 7:**
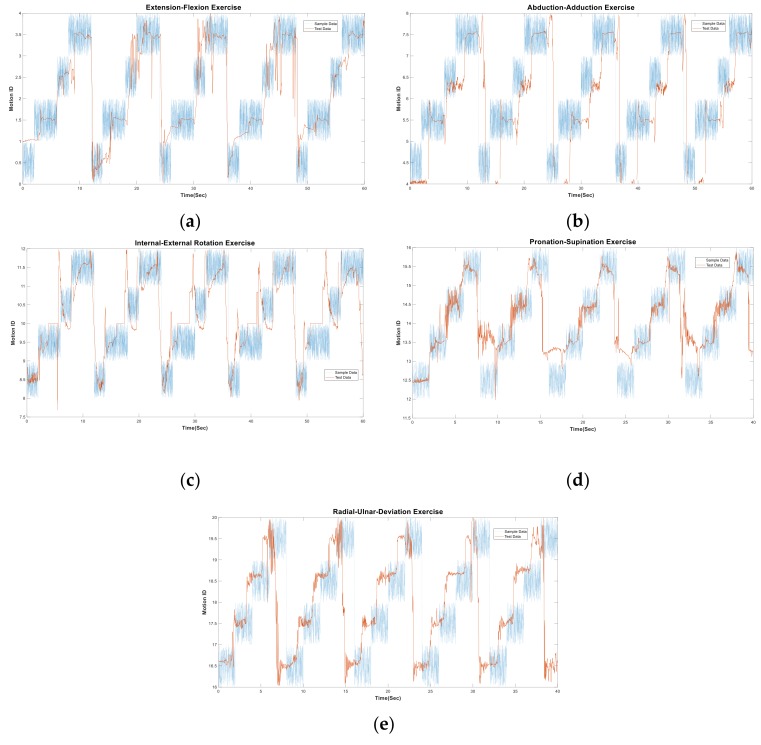
The inference diagrams of the test data versus the sampling schedule for the simple exercises: (**a**) flexion and extension, (**b**) abduction and adduction, (**c**) external and internal rotation, (**d**) supination and pronation, (**e**) ulnar and radial deviation.

The test datasets involve the same input features as the sample, and the output motion labels are inferred by the trained FIS. The sample motion labels were produced due to the exercise schedules for comparison. The inference diagrams of the five exercises are shown in Figure 7 to compare the inferred motion labels with the sample labels. Most of the joint motions could be recognized if the motion cycles satisfied the sampling schedules, and the inferred motion labels were plotted in the defined range. We observed that the exercises for the shoulder and wrist (e.g., Figure 7a,b,d,e) perform a better recognition effect than those for the elbow (e.g., Figure 7c), the latter being where the subject did not control the ext-int rotation of elbow in the expected ROM while changing motions. In comparison with the other four exercises, the elbow was not supported by a stable pivot to perfectly hold on the transverse plane while moving in the exercise.

With the inference diagram, the recognition performance can be visualized to track the exercise measurement. If the inferred motion labels are not plotted in the range of sampling motion labels, then the testing is either not obeying the assigned schedule or out of the limited motion range. Once the input value exceeds the limit of the FIS (i.e., an outlier), the inference is not available on the diagram. Thus, the subject who provides the sample dataset can obtain a personalized FIS to evaluate the test dataset in practice. The results above imply that the subject can be guided by an exercise schedule to rehab the joints with a trackable diagram. The diagram can be used to assessing whether the subject obeys the criteria or the exercise procedure needs further adjustment. The testing motion cycle, which was mostly fitted to the sampling schedule, was adopted from each simple exercise for evaluating the recognition rates in the next section.

### 3.2. Recognition Evaluation

The recognition rates of the chosen motions were evaluated by both the adaptable and quartile schemes as shown in Table 5. Both schemes include the outlier data that exceed the inference range (e.g., moving too fast and causing the angular velocity over the maximum value of sample data). The average recognition rates for *Exercises* A, B, and D, which perform steady motions according to the schedule, are from 0.809 to 0.927 due to the adaptable scheme; whereas that for the middle (50%) of the quartile scheme are from 0.879 to 0.992. For *Exercises* C and E, which did not involve stable movements, the rates ranged between 0.654 and 0.777 due to both schemes. This evaluation proves that the middle of the motion data are suitable for recognition.

We further checked on the details of the schemes for each motion. The scheme can offer the criteria of recognition for the unstable motions in the exercise such as the ext-int rotation in this study. For instance, we can define a five-grade criterion based on the different rates of recognition: Grade level 1 means 80% of data are recognized whereas level 4 is less than 20%, and level 0 is failed. In practice, if the grade level is larger than 3 (i.e., rate > 40%), then the motion of the subject can be confirmed as the acceptable recognition.

## 4. Implementation and Discussion

The simple ROM exercises of the PRR model can be applied to a hybrid mode including the union and complex exercises. The union exercise joins the simple exercises that are associated with the consistent FIS models. The complex exercise assembles several sub-exercises to train a unique FIS model, which involves all the features of the sub-exercises. Both exercise types were implemented in the self-development UHM system.

### 4.1. UHM Implementation

In the UHM system, the server layer can acquire full datasets of the subject for data training and generate the personalized FIS model; the gateway layer (i.e., mobile Apps can filter the sensor data and extract proper datasets for recognition according to the FIS. Then, the recognized motion labels can be illustrated on the trackable diagram for management. The subject can download the FIS to a personal smartphone and turn on the recognition mode of the App when starting a ROM exercise.

**Figure 8:**
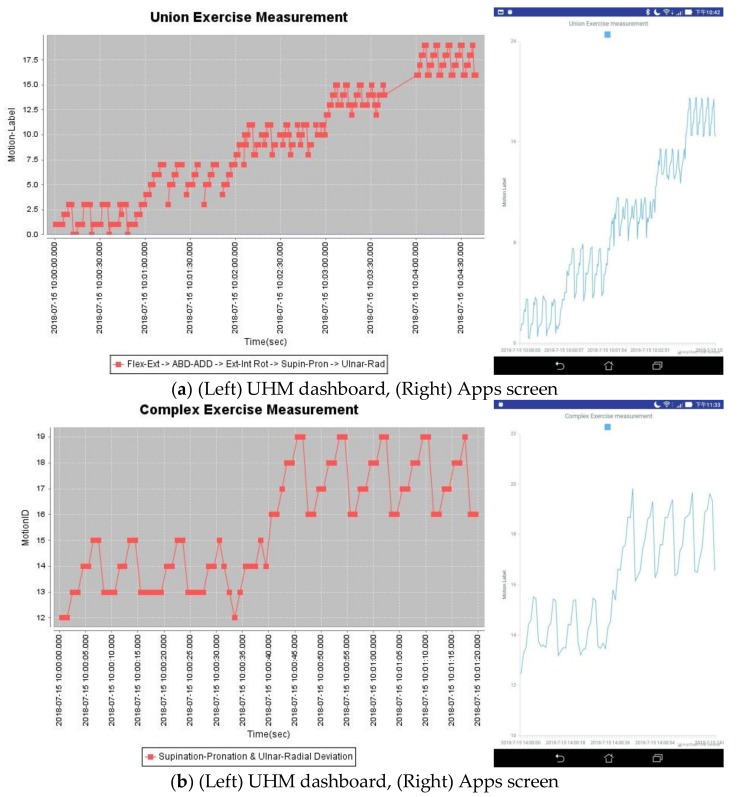
The trackable diagrams on the UHM dashboard and App screen for the ROM exercise application: (**a**) union exercise, (**b**) complex exercise.

The diagram of the exercise can be visually tracked on the web and mobile interface as shown in Figure 8, in which, the dashboard details each joint motion of the exercise over a set duration. Figure 8a displays the diagram tracking each motion in the union exercise as an example. The unrecognized motions can be noticed as outliers that were probably caused by incorrect posture or faulty movement. When processing the union exercise, the subject should select a FIS model with respect to the acting motion. All processed exercises are then joined together as a record set for uploading to the database. We then take the union exercise as an example to verify the scheme of true-false positive-negative rates, as shown in Table 6, for evaluating the implementation results. In which, the middle section of data in every second was retrieved for recognition. The scheme shows the estimators of true-positive, true-negative, false-positive, and false-negative for the five exercises. The averages of sensitivity, specificity, and accuracy are respectively about 0.81, 0.72, and 0.78 for this case.

For the case of complex exercise, the motion features of sub-exercises must be independent of the associate Fuzzy rules—i.e., there is no identical feature to control the different motions in the FIS model. We thus chose Exercise D and E as a complex exercise (i.e., pron-supin and ulnar-rad dev for the wrist joints). The sample datasets of both sub-exercises were merged for data training and their FIS models were combined as an initial FIS for tuning in the ANFIS. Notice that the unused features of the sub-exercise must be assigned by the virtual values to avoid confusing the data training process. For example by referring Table 2, both of Exercise D and E contain four features, i.e., “rANGVy”, “rANGx”, “rANGVz”, and “rANGz”. In training, the features “rANGVy” and “rANGx” are used by Exercise D but not E; thus, the virtual values (e.g., -999) are assigned to replace the measured values for these two features in Exercise E, and vice versa for the features “rANGVz” and “rANGz” used in Exercise D. In this case, the subject can select the available sub-exercises of the complex exercise with a FIS model. The tracking diagram can be managed as shown in Figure 8b.

### 4.2. Discussion

In general, the physiotherapist designs the program for patients to heal the injured joint with a simple exercise, and evaluates their practice in the rehabilitation room. We expect to transfer some programs from hospital to ubiquitous healthcare for a pilot study. The conventional body-motion measurement has difficulty in obtaining exercise identification with only a few sensors. For the aspect of UHM in rehabilitation, the exercise for joint movement limited in a ROM can be measured. If the patient can follow the therapy guidance, then a rigorous schedule can support a supervised machine learning scheme with labeled motions. We therefore consider the rehabilitation exercise by simplifying the complexity of recognition to enable the modeling in healthcare services. In the proposed PRR model, the subject must obey the physiotherapy exercise guide in order to create the sample data for a training process of ANFIS. The trained FIS approved by the test data could be applied to track rehabilitation records remotely in the UHM system. The advantages and limitations due to the implementation of the approaches are discussed below.

#### 4.2.1. Advantages

The labeled motion is feasible for recognizing scheduled exercises. Machine learning usually applies supervised algorithms for the recognition requirement. For the study of human activity recognition, unsupervised methods have been used to classify the clusters of various movement data in a labeling process [44]. With application to rehabilitation, the patient is guided by the designed exercise to rehabilitate the injured joint. Thus, the motions can be scheduled for the subject to produce the labeled sample data required for supervised machine learning.

The ANFIS is capable of training a personalized model for recognition. The initial FIS can be created due to the personal movement features such as sensor position, range of motion, etc. The fuzzy logic rules can be precisely controlled to enhance efficiency and the quality of data training in the ANFIS. Besides, the FIS can also be initialized for a specific exercise using the feature set with the proper MFs; then, the ANFIS can tune the FIS for a personalized model by fitting various sample datasets of the subjects who practice the same exercise.

The process of sensor data filtration can improve recognition in practical measurements. The proposed BLE-compliant sensor is able to stream raw data at up to 100 datapoints per second, which provides abundant sample datasets in the training procedure. The most popular algorithms can achieve a recognition rate of 85-95% for physical human activities, and this is enough for practical application [45]. Our approach evaluates a confidence interval of the motion to filter the proper data. The middle section in each scheduled motion period should be the potential data.

The PRR model details the customized motions of health informatics. The self-developed UHM system provides a trackable diagram, which is similar to the electrocardiography in telemedicine, on a Web-based and mobile interface. This approach allows assessment of specific motions in the physiotherapy exercise for the clinicians who want to understand more in details. For instance, the technology can be applied for the care in the self-monitoring of Parkinson’s diseases [46]. The motion variation and health information can be remotely monitored.

Comparing with the proposed model, the past studies had presented many machine learning algorithms for similar approaches [47,48,49]. Our previous work experienced the BPNN method to recognize different types of the frozen shoulder exercises [11]. We learned that the diverse machine learning methods can achieve good recognition models with the proper parameters. Thus the UHM system can be installed on a platform that offers comprehensive computing services with a variety of machine learning modules.

#### 4.2.2. Limitations

The proposed model has a limitation that the subject must wear the sensor at the same position for sampling and follow the identical exercise schedule. We thus consider two recognition schemes to help improve the implementation weakness seen in this study. In practical application, physicians trace a rehabilitation tendency rather than screen the exact movement of the patient. Within the developed UHM system, both the mobile and server sides can provide traceable diagrams in order to perform the recognition results. If not enough data points are captured, the system can report the possible reasons and suggest necessary adjustments according to the limitations.

Complex exercises cannot include identical features. The same feature can measure the various joint motions that are in the same ROM. For example, the features “rANGVx” and “rANGx” can be used for both of the flex-ext and abd-add exercises. However, they will be confused when used in the same FIS for a complex exercise. In addition, virtual values are necessary for the unused features of the sub-exercise in the data training process of ANFIS. In practice, the sub-exercise option is required for the mobile App to select the unused feature and replace the measured data with the virtual values. Therefore, we suggest wearing additional sensors on the limbs for the measurement when it comes to a complex exercise.

Highly irregular motions cause measurement outliers. The irregular motions in an exercise usually produce the unstable noises that implicate many ambiguous features in recognition. Most of the robot-assisted rehabilitation regimens can design a personalized program to support the UHM for self-monitoring management. If the patient performs the exercise but does not follow the schedule, the measurement is not reliable with the outliers on the tracking report. Thus, the patients probably need help from their healthcare provider for measurement at home. In clinical rehabilitation, the simple exercise is typically designed in the physiotherapy setting. The physicians can design a comprehensive PRR model involving necessary simple exercises for complicated rehabilitation.

In the present phase, the development is limited to the scheduled exercise for the patients who can follow the therapeutic design. In the next phase, we will consider the deep learning algorithms to train the practical measurement data. In which, the healthcare people can be involved to label the possible motions that are designed in the exercise but not exactly obeying the schedule. The future study is expected to improve the flexibility of the UHM system.

## 5. Conclusions Remarks

In this study, ANFIS was employed to generate a model of PRR, which can be utilized in a self-developed UHM system for physical therapy. The wearable sensor embedded with a BLE accelerometer and gyroscope chip can measure the motion data of physiotherapy exercises. Five simple joint motion exercises including shoulder, elbow, and wrist examples were designed and studied to demonstrate the modeling process. The subject wore the sensor-enabled wristband and moved the limbs following the scheduled motions to produce the sample and test data at a frequency of 100 data per second. Each motion dataset can be labeled with an arbitrary number in a defined range. Due to the sample datasets, the initial FIS was created by the steps of data preprocessing, feature visualizing, fuzzification, and Fuzzy logic rules. The ANFIS processed the data training cycles for tuning the FIS with the sample data to yield an optimal design. The trained FIS can estimate the test data based on the defuzzification process to infer the motion labels for recognition. The schemes of adaptive and quartile motion labels were used in evaluation. The average recognition rate was higher than 90% if the testing motions faithfully followed the sampling schedule. Thus, the middle portion of the motion datasets is suggested for recognition in practice. With implementation in the three-layer UHM system, the mobile App can retrieve the personalized FIS parameters to recognize the exercise and transport the records to the server for tracking. This approach finally contributes a feasible interface for the physicians to observe the trackable diagram on the server site and explore the rehabilitation motions in details. The UHM system can be integrated with the Internet of Things (IoT) for comprehensive health services in the future.

## Figures and Tables

**Table 1:** The ROM exercises and applicable joints of the upper limbs for rehabilitation.*.

Motion	Definition	Joints	Exercise
Flexion	move the limb along +Z axis on sagittal plane	shoulder, elbow, wrist	flexion-extension ^1^
Extension	move the limb along –Z axis on sagittal plane		
Abduction(ABD)	move the limb along +Z axis on frontal plane	shoulder	horizontal abduction-adduction ^2^
Adduction(ADD)	move the limb along –Z axis on frontal plane		
Rotation	rotate the limb or palm around Z axis on transverse plane	elbow, wrist	ext-int rotation ^3^, pronation-supination ^4^
Deviation	swing the wrist between radial and ulnar sides orthogonal to frontal plane	wrist	ulnar-radial deviation ^5^

* Motion abbreviation (ex: *italic word*) and motion-label range (ex: the range [a,b) means a≤ label <b); ^1^ Put arm down for extension: *rest-down*, [0,1); Extension: *ext*, [1,2); Hold arm on top for flexion: *rest-up*, [2,3); Flexion: *flex* [3,4); ^2^ Put arm down for adduction: *rest-low*, [4,5); Abduction: *ABD*, [5,6); Hold arm on shoulder level: *rest-half*, [6,7); *ADD*, [7,8); ^3^ Put arm inside: *rest-inside*, [8,9); External rotation: *ER*, [9,10); Hold arm outside: *rest-outside*, [10,11); Internal roation: *IR*, [11,12); ^4^ Put palm up: *rest-on*, [12,13); Pronation: *pron*, [13,14); Put palm down: *rest-under*, [15,16); Supination: *supin*, [16,17); ^5^ Bend the wrist on little finger side: *rest-right*, [17,18); Ulnar deviation: *ulnar-dev*, [18,19); Bend the wrist on thumb size: *rest-left*, [19,20); Radial deviation: *rad-dev*, [20,21)

**Table 2:** The candidate features for the ROM exercises in the study.*

Exercise	rANGVx	rANGVy	rANGVz	rANGx	rANGz
A. flex-ext Ex.	V			V	
B. abd-add Ex.	V			V	
C. ext-int rot. Ex.	V			V	
D. pron-supin Ex.		V		V	
E. ulnar-rad dev. Ex.			V		V

* Description of the features: rACCx, rACCy, rACCz: relative acceleration along three axes → start moving; rANGVx, rANGVy, rANGVz: relative angular velocity about three axes → in movement; rANGx, rANGy, rANGz: relative angle on three axes → limb position.

**Table 3:** The primary parameters of the trained-FIS for the flex-ext exercise.

**Input Features and Mamdani-Type MF (Vertex of Geometric Shape) ^1^**				
rANGVx Vertex Set Shape	MF1: i_rest [−120,−50,10] Triangle	MF2: i_flexion [−10,15,25] Triangle		MF3: i_extension [20,60,110] Triangle
rANGx Vertex Set Shape	MF1: i_rest_down [−180,−175,−140] Triangle	MF2: i_move [−150,−140.5,−19.9994,−9.98] Trapezoid		MF3: i_rest_up [−14.999,0.00043,15] Triangle
**Output Feature and Sugeno-Type MF (Coefficients of Linear Equation) ^2^**				
Motion Coefficient	MF1: o_rest_down [0.0077,0.1022,0.913]		MF2: o_flexion [0.0008,0.0002,1.5907]	
Motion Coefficient	MF3: o_rest_up [−0.1899,0.1779,33.5760]		MF4: o_extension [−0.0019,−0.0011,3.5074]	
Virtual Motion ^3^	null_1: [0.0181,−1.0772,−0.5743], null_2: [−0.0009,−0.0012,3.3421], null_3: [−0.0266,0.0076,4.2697], null_4: [−0.0118,0.0475,1.1785], null_5: [0.0011,−0.0427,−4.9702]			

^1^ For instance, [−120,−50,10] means the x coordinates of the left, middle, and right vertexes of the Triangle shape. ^2^ For instance, [0.0077,0.1022,0.913] presents a linear equation by z = 0.0077x + 0.1022y + 0.913, where x and y are the input features (i.e., rANGVx and rANGx). ^3^ All MFs in virtual motions are dummy in use but necessary for the complete logic rules.

**Table 4:** Fuzzy logic rules and corresponding MFs of features for the flex-ext exercise. ^1^

Feature	rANGVx	rANGx	Motion
Rule 1	i_rest	i_rest_down	o_rest_down
Rule 2	i_flexion	i_move	o_flexion
Rule 3	i_rest	i_rest_up	o_rest_up
Rule 4	i_extension	i_move	o_extension
Rule 5	i_rest	i_move	null_1
Rule 6	i_flexion	i_rest_down	null_2
Rule 7	i_flexion	i_rest_up	null_3
Rule 8	i_extension	i_rest_down	null_4
Rule 9	i_extension	i_rest_up	null _5

^1^ Note: Rule 5~9 are the dummy rules corresponding to the virtual motions null_1~null_5; the names of MFs are referred to Table 3. For example, the fuzzy logic of Rule 1: “If rANGVx is i_rest and rANGx is i_rest_down, Then the motion is o_rest_down”.

**Table 5:** Recognition results of the simple exercises for the test data in a cycle that can generally fit the sampling schedule. ^1^

Exercise	Joint Motion	Adaptable Scheme	Quartile Scheme		
			25%	50%	75%
**A.** **flexion-extension**	**rest_down**	0.9	1	1	1
	ext	0.765	0.225	1	1
	rest_up	0.51	0.4	0.55	0.283
	flex	0.96	0.941	0.966	0.975
	*average*	*0.809*	*0.642*	*0.879*	*0.815*
B. abduction-adduction	rest_low	0.97	1	0.967	1
	ABD	0.905	1	1	0.8
	rest_half	0.995	1	1	1
	ADD	0.863	0.866	1	1
	*average*	*0.927*	*0.967*	*0.992*	*0.95*
C. external-internal rotation	rest_inside	1	1	1	1
	ER	0.48	1	0.408	0.025
	rest_outside	0.52	1	0.383	0.033
	IR	0.723	0.714	1	0.874
	*average*	*0.654*	*0.929*	*0.698*	*0.483*
D. pronation-supination	rest_on	1	1	1	1
	pronation	0.975	1	0.933	0.95
	rest_under	0.905	1	1	0.7
	supination	0.78	1	1	0.627
	*average*	*0.915*	*1*	*0.983*	*0.819*
E. ulnar-radial deviation	rest_right	0.965	1	1	0.917
	ulnar_dev.	0.7	0.833	0.767	0.433
	rest_left	0.95	1	1	0.85
	radial_dev.	0.405	0.475	0.339	0.441
	*average*	*0.755*	*0.827*	*0.777*	*0.660*

^1^ Note: Rule 5~9 are the dummy rules corresponding to the virtual motions null_1~null_5.

**Table 6:** The verification scheme of true-false positive-negative rates for the union exercise. ^1^

Exercise No.	TP	FN	FP	TN	TPR	FPR	TNR	ACC
A	15	4	0	1	0.79	0	1	0.8
B	15	1	0	4	0.94	0	1	0.95
C	5	0	6	9	1	0.4	0.6	0.7
D	15	4	1	0	0.79	1	0	0.75
E	7	6	0	7	0.54	0	1	0.7
Average					0.81	0.28	0.72	0.78

^1^ Note: TP = true-positive data; FN = false-negative data; FP = false-positive data; TN = true-negative data; TPR(sensitivity) = true-positive rate = TP/(TP+FN); FPR(fall-out) = false-positive rate = FP/(FP+TN); TNR(specificity) = true-negative rate = TN/(FP+TN); ACC = Accuracy = (TP+TN)/(TP+FN+FP+TN);.

## Appendix A

Figure A1, Figure A2, Figure A3 and Figure A4 are the sampling data of input features for the *Exercises B* to *E*, respectively.

## Appendix B

Figure B1, Figure B2, Figure B3 and Figure B4 are the fuzzy sets with membership functions of FIS design corresponding to *Exercises B* to *E*, respectively.
